# Breast Cancer Hormone Receptor Status Determination from H&E-Stained Biopsy Images Using Pixel-Level Classifiers

**DOI:** 10.3390/cancers18132085

**Published:** 2026-06-27

**Authors:** Shuyang Wu, Ines P. Nearchou, Sandrine Prost, Jonathan A. Fallowfield, Hideki Ueno, Hitoshi Tsuda, Alastair Ironside, David J. Harrison, Timothy J. Kendall

**Affiliations:** 1 Centre for Inflammation Research, Institute of Regeneration and Repair, University of Edinburgh, Edinburgh EH16 4UU, UK; frank.wu@ed.ac.uk (S.W.); s.prost@ed.ac.uk (S.P.); jonathan.fallowfield@ed.ac.uk (J.A.F.); 2 Indica Labs, LCC, 8700 Education Pl NW, Bldg. B, Albuquerque, NM 87114, USA; inearchou@indicalab.com; 3 Department of Surgery, National Defense Medical College, 3-2 Namiki, Tokorozawa 359-0042, Japan; ueno_surg1@ndmc.ac.jp; 4 Department of Diagnostic Pathology, Seikei-kai Chiba Medical Centre, 1-7-1 Minami-cho, Chuo-ku, Chiba 260-0842, Japan; h-tsuda@seikei-kai.or.jp; 5 Department of Basic Pathology, National Defense Medical College, 3-2 Namiki, Tokorozawa 359-0042, Japan; 6 Directorate of Laboratory Medicine—Pathology Department, NHS Lothian University Hospitals Division, Western General Hospital, Edinburgh EH4 2XU, UK; alastair.ironside@nhs.scot; 7 School of Medicine, University of St Andrews, St Andrews KY16 9AJ, UK; david.harrison@st-andrews.ac.uk

**Keywords:** digital pathology, machine learning, hormone receptor status prediction, breast cancer, slide-level prediction

## Abstract

**Background:** Analysis of digital images of histopathological sections is increasing due to widespread adoption of fully digitised workflows and the greater availability of whole-slide scanners. Currently, hormone receptor status in breast carcinoma is assessed by pathologists scoring separate immunohistochemically stained sections. **Methods:** In this study, we employed pathologist-verified pixel-level annotations to train nested pixel classifiers capable of making case-level predictions of oestrogen receptor (ER), progesterone receptor (PR), and human epidermal growth factor receptor 2 (HER2) status directly from H&E-stained sections using biopsy cases alone. The model was evaluated on both an internal test set and an external international evaluation set from an institution in a different continent using different scanner hardware without the need for image normalisation. **Results:** In the internal test set, the models achieved AUCs of 0.8030, 0.7956 and 0.7488 for ER, PR, and HER2, respectively, with AUCs of 0.7008 and 0.7488 for ER and PR using an external cohort from an institution from which no cases were used for training. **Conclusions:** Our data highlight a potential strategy by which a pixel-based classifier, typically developed to quantify histological features within individual cases, could be used to make case/slide-level predictions but illustrate the challenges associated with this approach.

## Simple Summary

Digital scans of stained tissue sections are increasingly used by computational digital pathology tools to gain insights that cannot be provided by human pathologists. Tumour biopsies from patients with breast cancer are tested for the presence of receptors (ER, PR, HER2) by direct immunohistochemical staining of the receptors, assessed by pathologists, to determine treatment choices. We have used a digital platform using manual annotations to train tools that classify regions of tumour in haematoxylin and eosin-stained sections as receptor-positive or negative, and these are used to predict the receptor status of the case. Model training has been undertaken using a platform with a graphical user interface that needs no specialist computational skills to show how such tools might be used by biologists and pathologists without coding experience but the work also illustrates the challenges associated with this approach. The overall performance in test cases highlights the need to consider enriching training cases to include those from multiple laboratories to capture staining variation and to add greater numbers of rare histological subtypes, alongside extensive additional external validation, if development towards clinical application were to be pursued.

## 1. Introduction

Female breast cancer is the most common malignancy worldwide, with an estimated 2.3 million new cases annually that accounts for 16% of all cancer deaths [1]. Treatment strategies are largely determined by the expression of oestrogen receptors (ERs) [2] and progesterone receptors (PRs) and the copy number of the human epidermal growth factor receptor 2 (*ERBB2*) [3] gene [4,5,6] that encodes the HER2 protein. Although immunohistochemistry (IHC) is widely used to identify and quantify these tissue biomarkers, the process introduces reporting delays and incurs significant financial costs and its limitations have been appreciated for many years [7]. Furthermore, biases may arise from variations in laboratory protocols and the inherently subjective interpretation of stained sections by pathologists [8]. For example, in a multicentre study in 2020 with 5 pathologists scoring HER2 IHC, only a third of cases achieved complete score agreement between all pathologists [4].

Previous studies have demonstrated that an AI-guided method can considerably improve the interobserver agreement among different pathologists for such tasks [9,10]. In contrast to IHC, H&E staining is ubiquitous, with H&E-stained sections provided as the standard in all pathology laboratories. Whilst many publicly available datasets, such as The Cancer Genome Atlas (TCGA) [11] and CAMELYON Challenges [12,13], primarily feature resection data, biopsy cases are of greatest clinical significance because features, including hormone receptor status, determined from them guide neoadjuvant therapy and surgical planning. Resection cases include greater representation of lesional tissue, but tools trained on only resections cannot be assumed to be applicable to biopsy cases on which the real-world decisions are made. Consequently, the computational determination of hormone receptor status directly from H&E-stained biopsy sections offers considerable potential benefits, including reduced reporting times, lower costs, and minimised subjectivity.

With the rapid advancement of digital pathology (DPATH), deep learning platforms are increasingly being developed for the analysis of digital whole-slide images of both stained and unstained sections [14]. The HALO AI [15] platform is capable of complex tasks such as pixel classification, tissue and membrane segmentation, and cell phenotyping [16,17,18]; thus, it holds significant potential for taking this further to make case-level breast cancer hormone receptor status predictions.

**Figure 1:**
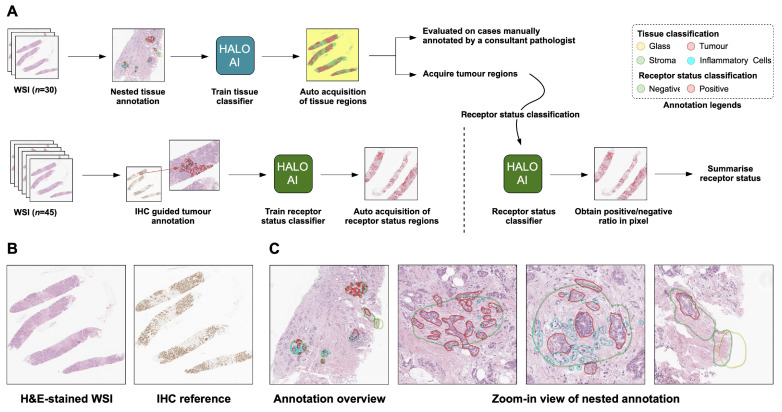
Overview of pipeline and annotation protocol. (**A**) Overview of the tissue classifier and receptor status classifier pipeline using HALO AI. (**B**) An example of an H&E-stained image and its corresponding IHC reference. (**C**) An example of an annotated image and high-power view of nested annotation.

In this paper, we use HALO AI to train classifiers for three tasks (ER, PR, and HER2 status classification) using an internal breast cancer cohort, evaluating performance on both an internal independent test set and an external evaluation set. We demonstrate that our classifiers can accurately perform tissue segmentation and automatically classify receptor status based on the tumour area at the pixel level. Furthermore, our work presents an accessible and viable approach for biomedical scientists, pathologists, and other clinicians without a computational background to train and apply pixel-level classifiers, generating slide-level classifications for, in this instance, hormone receptor status (Figure 1A).

## 2. Materials and Methods

### 2.1. Datasets and Ethics

The internal data were supplied by the NHS Lothian BioResource (Research Tissue Bank ethical approval from East of Scotland Research Ethics Services ref: 25/ES/0030) with research project approval SR2036, 10 August 2023). The internal study cohort consists of 491 biopsies of primary breast carcinoma from January 2018 retrieved from the clinical archive on the basis of pathology report alone; no curation or case selection based on the specific reported subtype of carcinoma or subjective slide quality was undertaken. Reported diagnostic composition of the cohort is shown in Table 1. Following routine institutional SOPs, biopsies were fixed in 4% buffered formalin for between 24 and 48 h. Blocks were sectioned at 3 μm for H&E staining.

Hormone receptor status was determined as part of routine clinical reporting by consultant pathologist assessment of IHC-stained sections or FISH for cases with IHC HER2 2+ results. The complete clinically reported case-level IHC receptor and FISH results for the cohort are visualised in Supplementary Figure S1A. Routine clinical ER IHC used Agilent (Dako) clone EP1 (Agilent Technologies, Glostrup, Denmark), dilution 1:30. Routine clinical PR IHC used Agilent clone PgR636 (Agilent Technologies, Glostrup, Denmark), dilution 1:200, stained on Leica BOND III platform (Leica Biosystems, Nussloch, Germany). Routine clinical HER2 IHC used Leica clone CB11, ready to use Oracle kit (Leica Biosystems, Nussloch, Germany). Whole-slide images of de-identified clinical H&E-stained sections were acquired using an Aperio GT450DX scanner (Leica Biosystems, Nussloch, Germany).

The external data for the study was approved by the Ethics Committee of the National Defense Medical College, Tokorozawa, Japan (Approval Nos. 4913, 11 January 2024, and 5009, 16 May 2024). The external validation cohort comprised H&E-stained pathology slides of core needle biopsy specimens obtained from 205 consecutive patients with primary breast cancer from January 2017 to December 2019 from the archives of the Department of Diagnostic Pathology, National Defense Medical College Hospital (NDMCH), Tokorozawa, Japan; no curation or case selection based on the specific reported subtype of carcinoma or subjective slide quality was undertaken. Reported diagnostic composition of the cohort is shown in Table 1. Following routine institutional SOPs, biopsies were fixed in 10% buffered formalin for between 24 and 48 h. Blocks were sectioned at 3 μm for H&E staining.

Hormone receptor status was determined as part of routine clinical reporting by consultant pathologist assessment of IHC-stained sections or FISH for most cases with IHC HER2 2+ results. The complete clinically reported case-level IHC receptor and FISH results for the cohort are visualised in Supplementary Figure S1B. Routine clinical ER IHC used Roche Diagnostics clone SP1 (Roche Diagnostics, Tokyo, Japan), dilution 1:100. Routine clinical PR IHC used Roche Diagnostics clone IE2, dilution 1:100. Routine clinical HER2 IHC used Roche Diagnostics clone 4B5 (Roche Diagnostics, Tokyo, Japan), diluted 1:100, stained on Roche Diagnostics Ventana BenchMark XT (Roche Diagnostics, Tokyo, Japan). Whole-slide images of de-identified H&E-stained sections were acquired using a NanoZoomer SQ scanner (Hamamatsu Photonics, Hamamatsu, Japan). Corresponding de-identified data for ER, PR, and HER2 status were provided by the Department of Diagnostic Pathology at NDMCH.

The hormone receptor status that was part of the original clinical pathology report was utilised to categorise the cases into binary classes: ER+/ER−, PR+/PR−, and HER2+/HER2−, respectively. For ER and PR, Allred scores were provided as part of the original clinical report; in accordance with clinical guidelines, we defined scores of 0 and 2 as negative, and scores of 3–8 as positive [19,20]. For HER2 classification, we defined scores of IHC 0, 1+, and 2+ without FISH-proven amplification as negative, whereas HER2 IHC 2+ cases with FISH amplification and all HER2 IHC 3+ cases were classified as positive. No data pre-processing or curation was applied to the whole-slide images.

### 2.2. Classifier Training

All classifiers were trained using whole-slide images (WSIs) of H&E-stained sections. No whole-slide image computational preprocessing was applied, and images were not cropped to human-defined regions of interest such that all the tissue from a single section from the clinical blocks was used. No images of IHC-stained sections were used for model training. Model training and testing was undertaken using HALO AI (version 3.6.4134.464).

#### 2.2.1. Tissue Classification

A tissue classifier was developed to categorise all pixels within the WSIs into one of five distinct classes: ‘background’, ‘stroma’, ‘tumour’, ‘inflammatory cell’, or ‘red blood cell’. The training set comprised 30 cases from the internal cohort, with manual pixel-level annotations performed to capture full morphological and staining heterogeneity (Figure 1B,C). Annotations were undertaken iteratively and reviewed by a consultant histopathologist (TJK) and an Associate Principal Scientist working in computational pathology (IPN). A DenseNet [21] was employed as the tissue classifier, trained at a resolution of 0.5 microns per pixel (mpp) and a minimum object size of $50\;\mu m^{2}$.

To evaluate the classifier’s accuracy in identifying tumour pixels, F1-scores of model predictions on tumour regions were compared against ground-truth manual annotations performed by a consultant pathologist. This evaluation was conducted on a test set comprising discrete 1280×1280 μm regions extracted from 10 randomly selected cases. Subsequently, in a nested classification approach, regions identified as ‘tumour’ were utilised for training the specific hormone receptor status classifiers.

#### 2.2.2. ER Classification

To train the ER status classifier, we manually annotated WSIs of H&E-stained sections from 45 cases (15 ER+ cases and 30 ER− cases) from the internal cohort. The annotations were made following the same protocol as for the tissue classifier, guided by the positive regions in the corresponding IHC images. As a lower ER score (i.e., 3–6) reflects lower intensity and positive cell proportion, which would affect the annotation accuracy, to accurately annotate the ER+ cases, ER score 8 cases that are defined as the highest intensity and positive cell proportion were used. Annotated regions of the H&E-stained images were cross-referenced with the corresponding region of the IHC reference images. For ER− cases, tumour in ER score 0 cases was annotated in the image of H&E-stained sections. A DenseNet was employed to be the ER status classifier and was trained with resolution of 0.5 mpp and minimum object size of $50\;\mu m^{2}$.

To evaluate the classifier’s performance, we utilised the remaining images from the internal cohort alongside the external evaluation cohort. The internal test set comprised 442 cases with known ER status (394 ER+, 48 ER−), while the external evaluation set consisted of 205 cases (181 ER+, 24 ER−). In the tissue classification stage, both datasets were first processed by the tissue classifier to identify tumour regions. The ER status classifier was then applied exclusively to these predicted tumour areas. The final output of this nested approach was the proportion of tumour pixels classified as ER positive versus ER negative.

#### 2.2.3. PR Classification

The training schema and parameters for PR followed the same protocol as ER status classifier. For evaluation of PR status classification, the same sets of slides were used with the internal test cohort comprising 336 PR+ and 106 PR− and the external evaluation set of 165 PR+ and 40 PR− cases.

#### 2.2.4. HER2 Classification

To train the HER2 status classifier, we manually annotated 45 cases (15 HER2+ and 30 HER2−) from the internal cohort. The training schema and parameters follow the same protocol as ER and PR status classifiers.

For evaluation of HER2 status classification, the internal test set comprised a total of 443 cases including 37 HER2+ cases and 406 HER2− cases, with HER2 2+ cases without FISH results removed. For the external evaluation set, 205 cases were employed after removing those HER2 2+ cases with unknown FISH results and consisted of 26 HER+ cases and 179 HER− cases.

### 2.3. Generation of Slide-Level Hormone Receptor Status from Pixel Classification Results

The proportion of tumour area classified as hormone receptor-positive was utilised to derive a specific cut-point for defining case-level hormone receptor status. To statistically validate the efficacy of the positive proportion as a predictive indicator, we randomly partitioned the testing dataset into two 6:4 subsets, maintaining balanced receptor status categories. 60% of the data functioned as a pseudo-training set to identify the optimal cut-point in the hormone receptor proportion metric that most accurately predicted receptor status. The remaining 40% served as a pseudo-testing set to evaluate performance using the determined cut-point. We employed the ‘maxstat’ R package (2024.04.1+748) to ascertain the optimal cut-point within the pseudo-training set and the cut-point was subsequently used to compute the Area Under the Curve (AUC) and F1-score on the pseudo-testing set. This entire procedure was applied for both the internal and external test set and repeated five times to simulate a five-fold cross-validation scenario.

The cross-validation approach used throughout follows the schema used by previous works. The data were split into 5 different parts (‘folds’), 3 folds used as the training set for each model, 1 fold as the validation set, and 1 held-out fold as the test set to determine model performance. This is repeated 5 times such that each fold is used as a validation set once only and the predictive performances are averaged. The training, validation, and test sets each time are independent from one another [22,23,24].

### 2.4. Evaluation Metrics

We used the F1-score to measure the performance of the tissue classifier on classifying tumour areas. For the hormone receptor classifiers, Area Under the Curve score (AUC) and macro F1-score are used to evaluate the classification performance.

### 2.5. Review of Cases with Discrepant Classifier Predictions

For each receptor prediction, cases from the external validation cohort that were classified incorrectly were manually reviewed by a consultant histopathologist (TJK) to determine if any consistent features were evident that may account for the prediction failures. This assessment was synthesised to create a narrative review.

## 3. Results

### 3.1. Overall Reported Histopathological Description of the Internal and External Cohorts

The overall composition of the internal and external cohorts based on the clinically reported tumour type is shown in Table 1. The reported ER IHC score, PR IHC score, HER2 IHC score, and FISH *ERBB2* amplification status, when tested, are visualised at case level in Supplementary Figure S1.

### 3.2. Pixels of Tumour Cells Are Accurately Identified by the Trained Tissue Classifier

**Figure 2:**
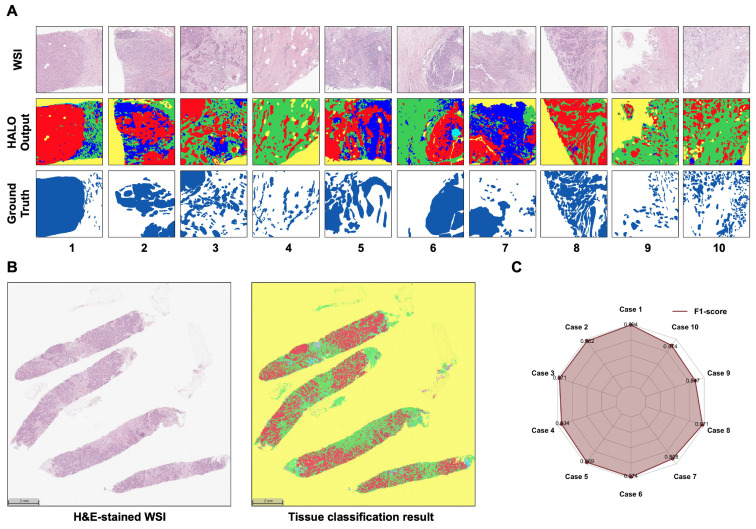
Results of the tissue classifier on manual patch test set, which comprise discrete regions in 1280×1280 μm under 40× magnification from 10 randomly selected cases. (**A**) Visualised results comparing original WSI vs. HALO output vs. ground truth annotation in tissue classification. (**B**) An example of an H&E-stained image and its tissue classification map provided by HALO. (**C**) F1-scores for the tumour area in test patches.

The tissue classifier accurately identifies tumour cell pixels in breast cancer biopsy H&E-stained images (Figure 2) when compared with pathologist manual annotations. The mean F1-score for the individual manually annotated test patches reaches 0.9393.

### 3.3. ER, PR, and HER2 Slide-Level Status Can Be Derived Using Pixel-Level Classifiers Applied to Images of H&E-Stained Sections

**Figure 3:**
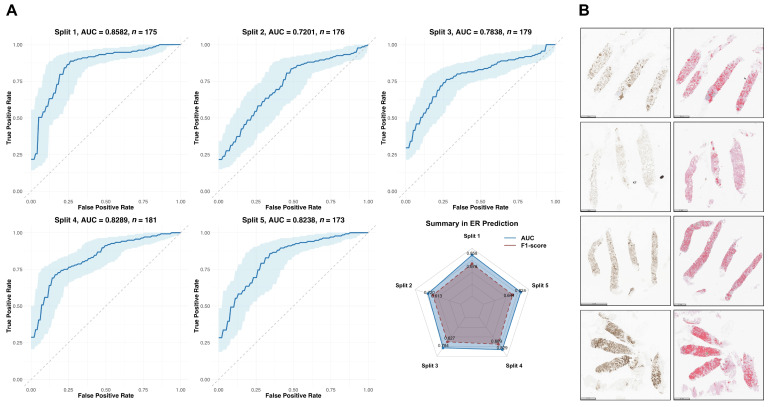
ER status classification results on internal independent test set. (**A**) The Receiver Operating Characteristic (ROC) curves with the corresponding AUC scores and F1-scores. (**B**) The visualised examples for ER+ cases.

**Figure 4:**
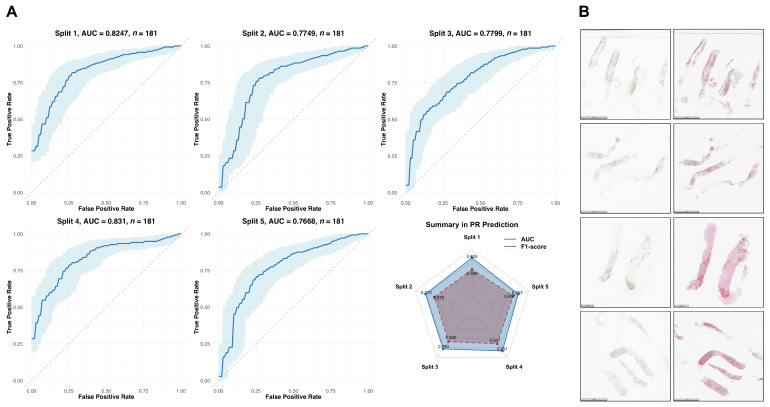
PR status classification results on internal independent test set. (**A**) The Receiver Operating Characteristic (ROC) curves with the corresponding AUC scores and F1-scores. (**B**) The visualised examples for PR+ cases.

**Figure 5:**
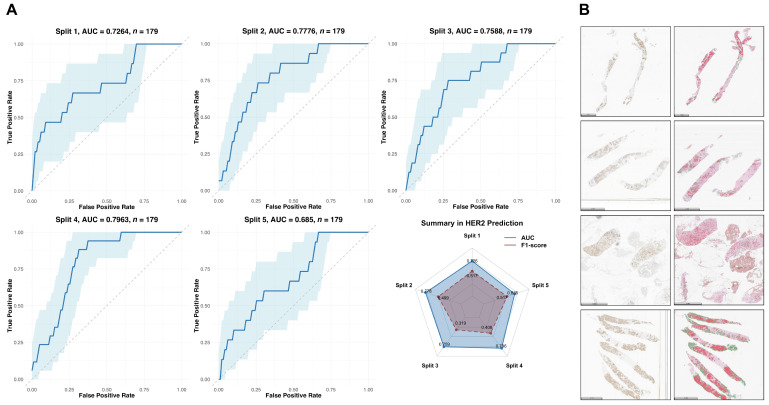
HER2 status classification results on internal independent test set. (**A**) The Receiver Operating Characteristic (ROC) curves with the corresponding AUC scores and F1-scores. (**B**) The visualised examples for HER2+ cases.

**Figure 6:**
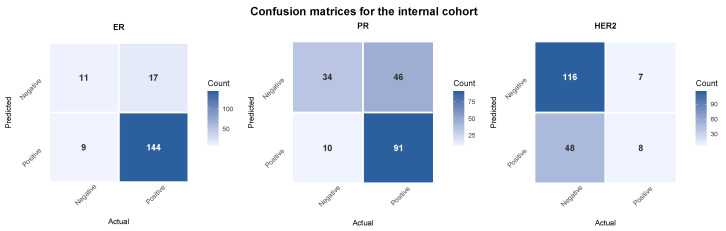
Confusion matrices for ER, PR, and HER2 status classification on the internal cohort. The best split in five-fold cross-validation on each task is adopted for the corresponding confusion matrix.

Tumour pixels were classified as ER+ or ER− and the defined ER+ proportion cut-point applied to the ER pixel classifier output to provide a slide-level ER status that was evaluated against ground truth (Figure 3). An average AUC of 0.8030 and an F1-score of 0.6502 were achieved in the internal test set. Furthermore, the classifier generates visualisation maps identifying predicted ER-positive tumour areas (Figure 3B) that allow subjective inspection. Similarly, slide-level determination of PR (Figure 4) and HER2 (Figure 5) status was achievable using the same pixel classification approach. In the internal test set, the PR classifier yielded an average AUC of 0.7956 and an F1-score of 0.6098, while the HER2 classifier attained an average AUC of 0.7488 and an F1-score of 0.452. Confusion matrices showing the predictions of internal cohort cases are shown in Figure 6.

### 3.4. Slide-Level Hormone Receptor Status Prediction from Pixel Classifiers Can Be Applied to Images from a Different Institution Acquired Using Different Scanner Hardware Without Additional Training

**Figure 7:**
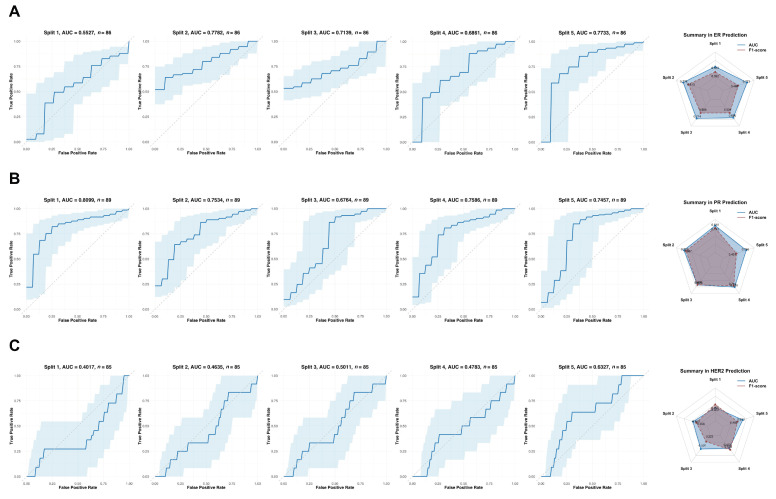
Results for ER, PR, and HER2 status classification on the external evaluation set. (**A**–**C**) show the Receiver Operating Characteristic (ROC) curves with the corresponding AUC scores and F1-scores for ER, PR and HER2 status classification, respectively.

**Figure 8:**
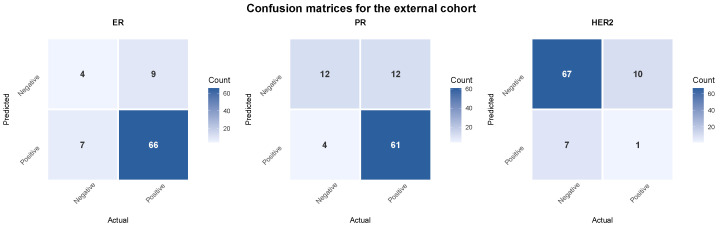
Confusion matrices for ER, PR, and HER2 status classification on the external cohort. The best split in five-fold cross-validation on each task is adopted for the corresponding confusion matrix.

The potential generalisability of slide-level hormone receptor status predictions based on pixel classifiers was assessed using an external evaluation set. Cases were selected from an independent hospital in Japan, using the sections stained in the allied pathology laboratory in the same institution, and imaged using different scanner hardware (Hamamatsu NanoZoomer, Hamamatsu Photonics, Hamamatsu, Japan). No pre-processing or stain normalisation was applied prior to classification. The average AUC and F1-scores for the three tasks are presented in Figure 7. ER and PR classifications achieved AUC scores of 0.7008 and 0.7488, with corresponding F1-scores of 0.5012 and 0.6496. These results show a reduction in performance compared with that seen with the internal independent test set. The HER2 classification exhibited an AUC of 0.4955 and an F1-score of 0.4148, indicating greater performance degradation in this complex task when applied to the external cohort. Confusion matrices showing the predictions of external validation cohort cases are shown in Figure 8.

### 3.5. No Consistent Histopathological Features Are Common Across Cases Incorrectly Predicted by Classifiers

For each receptor classifier, cases incorrectly predicted were manually reviewed. The incorrectly predicted cases were not those containing limited amounts of invasive tumour compared with correctly predicted cases. Incorrectly predicted cases did not obviously contain more necrosis or inflammation. The exact diagnosis of cases from the external validation cohort that were correctly and incorrectly predicted for each biomarker in the confusion matrices in Figure 8 are shown in Supplementary Table S1. Examples of correctly and incorrectly predicted cases for ER, PR, and HER2 from the same splits are shown in Supplementary Figure S2.

## 4. Discussion

We have demonstrated a strategy by which a GUI-based digital pathology tool, requiring no prior programming or command line experience and typically used to quantify features within individual slides, can be used to train classifiers via a nested annotation approach. These classifiers are used to generate slide-level predictions directly from H&E-stained biopsy sections for properties typically determined via immunohistochemistry. However, although we present an approach for training a tool using a code-free platform to make case-level predictions using an exemplar clinical application, the performance on internal and external test sets remains below that required for clinical application; to take such tools through the regulatory process would require significant further development and time, and the final performance would need to be higher than presented in this exemplar and proven to be robust across additional external cohorts.

A number of study limitations could be specifically addressed to improve performance, reflecting more general challenges in computational pathology studies. The training internal cohort was almost exclusively ductal carcinomas of no special type, so generalisability of the trained classifiers to carcinomas of special type is unlikely, and, indeed, some of the limited number of special type cases included in the external validation cohort as representative of real-world case mix were misclassified. Any attempts to continue development of this classification method further, or projects using fully computational methods, require either significant enrichment of the training set with more cases of defined special types of breast cancer or an explicit decision to develop a tool for exclusive use on cases histologically diagnosed as ductal carcinoma of no special type. Such a decision is likely to be pragmatic and based on the number of cases of special-type cancers available for training.

While no explicit colour calibration requirement was used in this study, consistent staining and scanner calibration may be advisable to ensure reproducibility across sites, and the reduced performance in the external validation set may, in part, reflect that. Although human readers of slides are quickly able to compensate for differences in staining, even subtle differences have been shown to significantly reduce computational model performance. For example, Zhou et al. [25] successfully trained a deep learning model to predict brain metastasis from patients with non-small cell lung cancer (NSCLC) from H&E images. However, it was shown by the follow up work [26] that even staining variation introduced by staining new sections from the same blocks in the same laboratory only 8 months later significantly reduced model performance. Many computational methods for stain normalisation have been explored to attempt to address this problem [27], although such methods may still be unable to fully compensate for staining variation, as illustrated by the failure of the generative adversarial network normalisation approach [26]. The effect of staining variation may be mitigated by the use of very large datasets including WSIs from multiple different laboratories to effectively capture the full range of staining. Such approaches underpin the more recent training of newer foundation models such as CONCH [28], UNI [29], GigaPath [30] and Virchow [31], which uses multi-centre pan-tissue datasets for pre-training. Any development of classifiers that aspire to generalisability, such as the exemplar presented here, would benefit from either training using images that reflect the range of staining variation seen in sections from the laboratories that may use the final tool and/or stain normalisation strategies.

Alternative fully computational methods [32,33,34,35,36,37] rely on hyper-parameter tuning and complex coding pipelines, and do not leverage expert, tissue-level annotations during training. Such computational methods may, however, offer performance advantages in training slide/case level prediction models. In contrast, an integrated software solution with a GUI that does not require model architecture customisation and hyper-parameter tuning is more accessible for clinicians without a programming background. This is in line with recent efforts to move towards code-free implementation of computational pathology tool development [38]. Furthermore, the visual maps generated by these classifiers introduce interpretability, allowing pathologists or biologists to examine the areas of the tumour predicted to be biomarker-positive, thereby bridging the gap between black box algorithms and clinical intuition. The choice of methodology by investigators should balance the increased accessibility to biologists of GUI-based systems against the potential performance benefits of fully computational methods, such as those using multiple-instance learning, that require coding skills and do not use tissue annotations, but learn features associated with a case- or slide-level property. The choice of tool is likely to be highly task-dependent, and it may be advantageous for investigators to explore such complementary methods agnostically rather than the choice being solely based on investigator preference as a function of expertise.

The determination of HER2 receptor status from H&E images proved less accurate than that of ER and PR status, particularly within the external validation set. This suggests that the overall cellular morphology utilised for training and prediction may be more strongly influenced by nuclear transcription factors than by the morphological changes associated with HER2 positivity or *ERBB2* gene amplification. This aligns with recent findings that the absence of biologically interpretable morphological features in H&E-stained slides limits their utility for the quantitative prediction of HER2 expression levels compared to nuclear biomarkers [39]. Furthermore, the images of H&E-stained sections used for training annotations and IHC reference images are not co-registered in the current scheme; fully co-registered IHC references may allow automatic individual cell annotations to be transferred to H&E images to further improve the annotation accuracy and classifier performance, while virtual staining paradigms are also potentially available and of interest for further research [40,41]. By design, a classifier able to infer hormone receptor status from tumour cells in H&E-stained sections will leverage occult features present in the images but not cytological or histological features that are specific to IHC-stained sections; such features would be interpretable by a pathologist reporting the IHC but are not incorporated into this H&E-only model. The real-world nature of the datasets used meant that a small number of HER2 IHC 2+ cases in the external validation did not have available ISH status, i.e., lacked a ’ground truth’, so these cases were excluded for this hormone receptor.

HALO/HALO AI assumes the use of standard diagnostic whole-slide scanners (∼20×–40×), with no strict minimum or maximum resolution specified, and the platform supports a broad range of WSI formats (e.g., SVS, NDPI, MRXS, OME-TIFF, DICOM), enabling cross-site use. One additional advantage to classification tools developed using this platform is the potential for integration into HALO AP, an enterprise digital pathology platform that has FDA clearance for clinical use, thus representing a clearer potential line-of-sight to clinical application than open-source tools. By using only biopsy cases, our experimental design aligns with clinical practice, given that results from biopsies determine breast cancer treatment decision making [19]. Directly determining biomarker status from H&E-stained images significantly reduces laboratory work and time, removes human biases and subjectivity [36,42], and has been shown to correlate with downstream clinical prognosis [43].

## 5. Conclusions

This study demonstrates that a tissue annotation-based approach, utilising an accessible, coding-free GUI-based digital pathology platform, can be used to derive slide/case-level predictions from pixel classifications but illustrates the challenges of using this approach that must be addressed in order to reach clinical-grade performance.

## Figures and Tables

**Table 1:** Diagnostic composition of the internal and external cohort.

Cohort	Internal	External
Ductal carcinoma, no special type	489	178
Mucinous adenocarcinoma	1	12
Tubular adenocarcinoma	1	0
Invasive lobular carcinoma	0	10
Carcinoma with apocrine differentiation	0	2
Metaplastic carcinoma	0	2
Invasive micropapillary carcinoma	0	1
Total	491	205

## Data Availability

The internal dataset can be accessed by application to the NHS Lothian BioResource.

## Supplementary Materials

The following supporting information can be downloaded at: https://www.mdpi.com/article/10.3390/cancers18132085/s1, Figure S1: Overview of case-level receptor scores in the internal and external cohorts. (A) Alluvial plot showing the clinically reported ER IHC, PR IHC, HER2 IHC, and *ERBB2* FISH results in the internal cohort. (B) Alluvial plot showing the clinically reported ER IHC, PR IHC, HER2 IHC, and *ERBB2* FISH results in the internal cohort; Figure S2: Examples of correctly and incorrectly predicted cases from the external validation cohort for ER, PR, and HER2. Haematoxylin and eosin stain, scale bars 200 microns. Table S1: The exact diagnosis of cases from the external validation cohort that were correctly and incorrectly predicted for each biomarker in the confusion matrices in Figure 8.
