# Supplementary material for: Breast Cancer Hormone Receptor Status Determination from H&E-Stained Biopsy Images Using Pixel-Level Classifiers
Source: Cancers (Basel). 2026 Jun 27;18(13):2085. doi: 10.3390/cancers18132085 (PMC13360198; doi:10.3390/cancers18132085)
Supplement: Supplementary file 1 [file cancers-18-02085-s001.zip › supplementary_figures.pdf]

Breast Cancer Hormone Receptor Status Determination from H&E-stained Biopsy Images using Pixel-level Classifiers (Supplementary Materials)

A

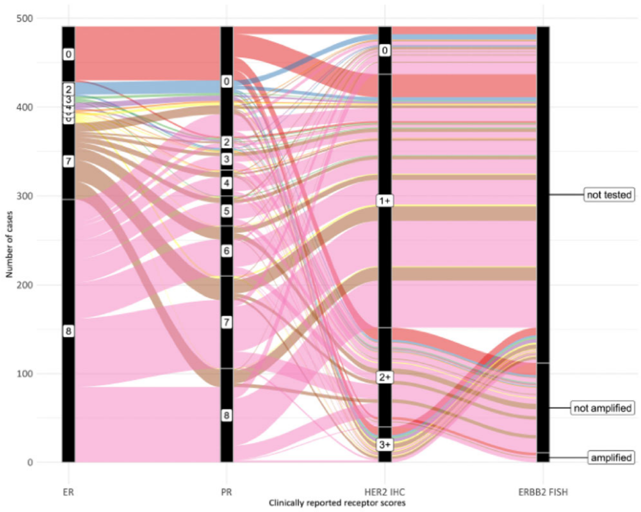

B

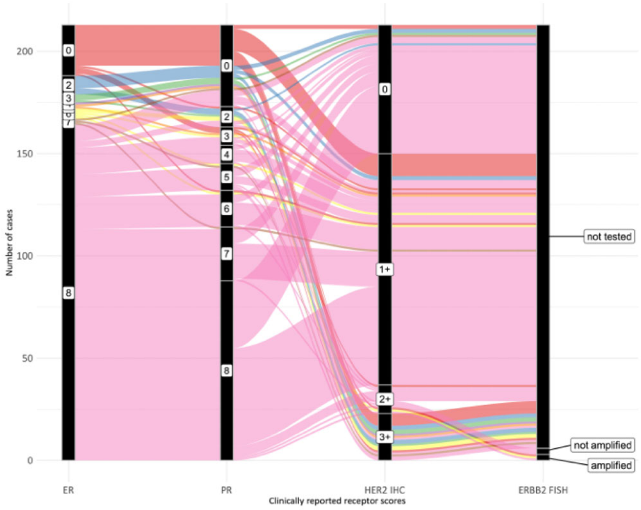

Supplementary Figure S1: Overview of case-level receptor scores in the internal and external cohorts. (A) Alluvial plot showing the clinically reported ER IHC, PR IHC, HER2 IHC, and ERBB2 FISH results in the

internal cohort. (B) Alluvial plot showing the clinically reported ER IHC, PR IHC, HER2 IHC, and ERBB2 FISH results in the internal cohort.

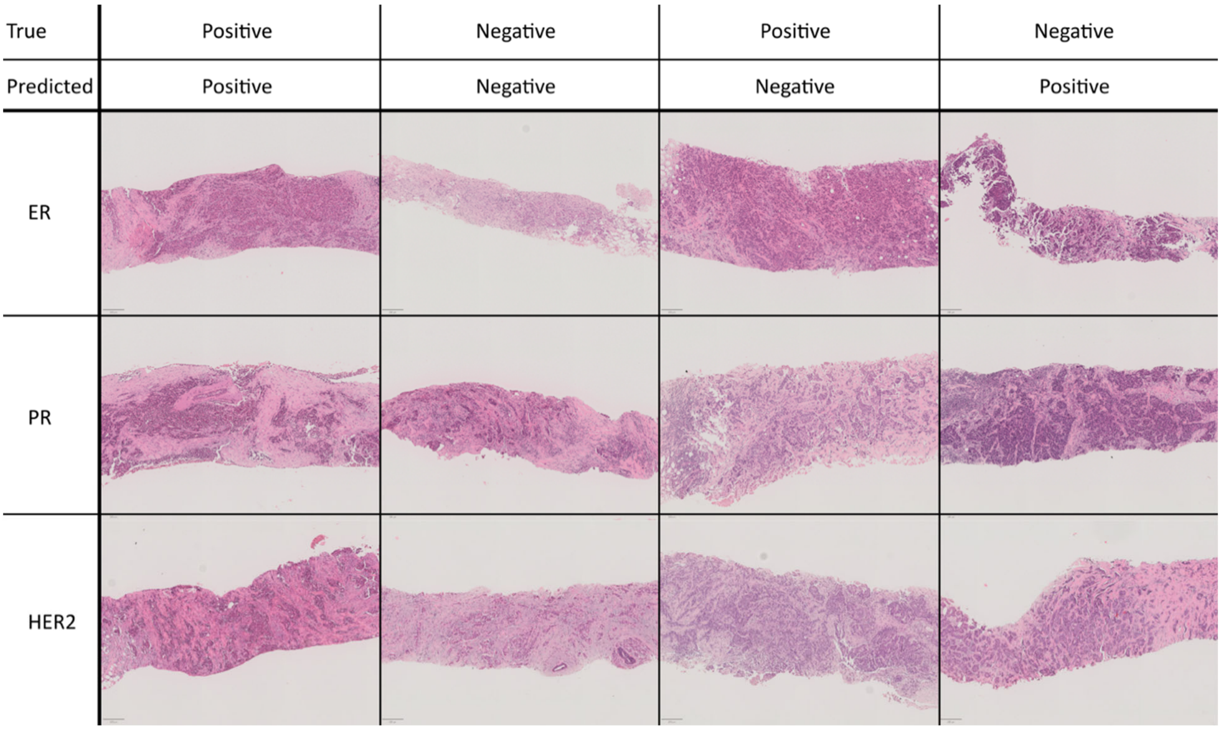

Supplementary Figure S2: Examples of correctly and incorrectly predicted cases from the external validation cohort for ER, PR, and HER2. Haematoxylin and eosin stain, scale bars 200 microns.
